# Deep learning-based detection and viability assessment of *Eimeria* oocysts

**DOI:** 10.1016/j.psj.2026.106419

**Published:** 2026-01-10

**Authors:** Hyeon W. Park, Matthew J. Valente, Valsin Fournet, Benjamin M. Rosenthal, Mark Jenkins, Asis Khan, Nitin Nitin

**Affiliations:** a Department of Food Science and Technology, University of California-Davis, Davis, CA 95616, USA; b Animal Parasitic Diseases Laboratory, Agricultural Research Service, US Department of Agriculture, Beltsville, MD 20705, USA; c Department of Biological and Agricultural Engineering, University of California-Davis, Davis, CA 95616, USA

**Keywords:** Artificial intelligence, Convolutional neural network, Parasite, Coccidiosis, Viability classification

## Abstract

Coccidiosis, caused by *Eimeria* species, is a significant disease affecting the poultry industry worldwide, leading to substantial economic losses due to reduced flock performance. Effective vaccination strategies require the precise quantification of the dosage of viable *Eimeria* oocysts to induce immunity in young chicks without causing disease. However, current methods for determining oocyst viability rely on sophisticated equipment and are not effective for routine monitoring. Recently, we documented the presence of granular structures exclusively in dead oocysts using high-resolution microscopic imaging. Hence, this study aimed to develop a simple, cost-effective approach using deep learning-based models to distinguish viable from non-viable *Eimeria* oocysts using morphological features, including the presence/absence of granular structures. Phase-contrast (PC), differential interference contrast (DIC), and brightfield (BF) imaging were employed to capture *E. acervulina* oocysts. The performance of a deep convolutional neural network based on the YOLOv7 architecture was evaluated for viability detection. Results indicated that the model trained with PC images outperformed those trained with DIC and BF, achieving overall precision and recall of 93.1 % and 91.2 %, respectively. Further dataset refinement, including class-specific labeling for sporulated, unsporulated, and dead oocysts, enhanced model performance, achieving an overall precision and recall of 99.1 % and 99.1 %, respectively. Cross-species evaluation of the method demonstrated that the model trained on *E. acervulina* generalized well to *E. tenella*, achieving 100 % overall precision and 98.1 % recall without additional training, whereas initial cross-species performance for *E. maxima* was substantially lower (43.5 % of overall recall), likely due to its larger oocyst size, but exceeded 95 % accuracy after fine-tuning with an *E. maxima*-specific dataset. This study highlights the potential of deep learning approaches to provide a practical, rapid, and reliable method for evaluating *Eimeria* oocyst viability, contributing to improved vaccine formulation and better coccidiosis management in the poultry industry. This proof of principle may also find application in assessing the viability of related parasites, such as *Cyclospora cayetanensis,* that pose a risk to human health and food safety.

## Introduction

Coccidiosis, caused by various species of the protozoan parasite *Eimeria*, is a major disease affecting the global poultry industry, which leads to significant economic losses and challenges in flock management (Cunha et al., 2020; Shivaramaiah et al., 2014; Swaggerty et al., 2015). The disease results in reduced weight gain, poor feed conversion, and increased susceptibility to other infections, even in subclinical cases (Swaggerty et al., 2015). The financial impact of coccidiosis is substantial, with estimated losses exceeding $800 million USD annually in the United States and $3 billion USD worldwide (Sharman et al., 2010; Shivaramaiah et al., 2014; Swaggerty et al., 2015). Decreased flock performance accounts for approximately 70 % of these losses, including reduced weight gain and poor feed conversion; prevention and control measures account for the remainder (Swaggerty et al., 2015).

Traditionally, producers rely on anticoccidial drugs, including ionophores and chemical coccidiostats, to mitigate disease (Cervantes, 2015; Williams, 2002; Beer et al., 2018). However, legislative restrictions and consumer preferences limit antimicrobial use in animal production, impelling a shift toward non-chemical methods (such as vaccination) (Cervantes, 2015; Gaghan et al., 2022; Zaheer et al., 2022). Vaccination involves administering low doses of sporulated, infective *Eimeria* oocysts to young chicks, aiming to induce protective immunity during the early weeks of life. Avoiding disease while priming effective immune responses necessitates precise control of the dosage of viable oocysts (Beer et al., 2018; Gaghan et al., 2022; Zaheer et al., 2022).

Accurate quantification of viable oocysts therefore, constitutes a critical challenge for consistent vaccine formulation (Jenkins et al., 2013; Snyder et al., 2023). Various factors influence oocyst viability, including storage duration and conditions. Over time, stored oocysts lose viability (Jenkins et al., 2023). Photochemical dyes, such as propidium monoazide and ethidium monoazide, have been used with PCR to evaluate membrane integrity and viability of parasites (Liang and Keeley, 2012; Rousseau et al., 2019). Flow cytometry can assess viability by detecting fluorescence properties of dyes, or by detecting autofluorescence of oocyst walls (Adams et al., 2022; Dixon et al., 2005; Jang et al., 2014; Robinson et al., 2023). Such methods require sophisticated equipment and specialized personnel, hindering their use for routine evaluation of *Eimeria* oocyst viability on farms. Thus, there is a pressing demand for simple, rapid, and cost-effective methods that the poultry industry can easily adopt for regular monitoring of viable *Eimeria* oocysts, ensuring consistent vaccine formulation and preventing infection from external sources.

In addition to its impact on poultry health and productivity, *Eimeria* serves as an important surrogate for *Cyclospora cayetanensis*, a related protozoan pathogen responsible for numerous foodborne outbreaks (Giangaspero and Gasser, 2019; Gutierrez et al., 2024; Tucker et al., 2022; Yeager et al., 2024). Of the more than 20 known species in the genus *Cyclospora* genus, *C. cayetanensis* is the only one known to infect humans (Dubey et al., 2022; Giangaspero and Gasser, 2019; Ortega et al., 1994). The lack of successful *in vitro* or *in vivo* propagation techniques for *Cyclospora* significantly hampers the advancement of effective detection and control strategies. Moreover, the limited availability of oocysts presents a hurdle in elucidating their maturation and biological processes (Dubey et al., 2022; Giangaspero and Gasser, 2019). This limitation impels the need for novel tools to detect viable pathogens in fresh produce and environmental samples, to evaluate the efficacy of hygienic interventions and disinfection procedures, and to advance experimental models to study this costly, emerging pathogen. Fortunately, a wealth of data from natural and experimental *Eimeria* infections and comparative genome analysis confirm that *Eimeria* and *Cyclospora* share conserved gene families, life-history traits, and developmental characteristics (Cinar et al., 2015; Dubey et al., 2022; Liu et al., 2016; Tang et al., 2015; Tucker et al., 2021). Consequently, *Eimeria* represents a promising surrogate for developing and evaluating improved methods for detection, diagnosis, and outbreak tracing of *Cyclospora* (Tucker et al., 2022). Using *Eimeria* as a surrogate to develop a rapid, sensitive, specific, and robust assay to diagnose parasite contamination and to test the presence of viable protozoan pathogens, particularly *Cyclospora*, will directly benefit producers and distributors of fresh produce and regulatory agencies charged with protecting public health.

Here, we sought to develop a simple and cost-effective method to evaluate oocyst viability by leveraging deep learning trained on morphological differences. Recent advancements in deep learning-based image analysis have shown great promise in the detection and classification of various microorganisms (Kang et al., 2024; Kim et al., 2021; Ma et al., 2023; Park et al., 2025a; Park et al., 2025b). For example, Kang et al. (2024) developed a 3D convolutional neural network model to classify different pathogenic bacteria using hyperspectral microscopic imaging. Ma et al. (2023) employed a standard plate-based cultivation method to form bacterial microcolonies within 3 hours and then classified various bacterial species using the YOLOv4 model. Yi et al. (2023) created an AI-biosensing framework that precisely detected *Escherichia coli* in real-world water samples by utilizing phage-induced lysis. Additionally, Park et al. (2025a) developed a deep learning model incorporating convolutional neural networks and generative adversarial networks to classify different yeast species in foods based on the morphological features of their microcolonies. The successful classification of bacteria and yeast species suggest promise for applying these methods to parasites such as oocysts of *Eimeria spp*.

To address the challenge of evaluating the viability of *Eimeria* oocysts in the poultry and food industries, we aimed to develop a simple and cost-effective approach using deep learning techniques. Recent high-resolution microscopic examinations have identified granular structures in dead oocysts that autofluorescence under UV exposure, which significantly increases the overall autofluorescence in these cells (Valente et al., 2025). We utilized this enhancement of autofluorescence intensity as a basis to distinguish live oocysts from dead ones using a Fluorescence-Activated Cell Sorting (FACS) system. We validated this distinction by demonstrating infectivity in chickens with live oocysts while observing minimal shedding with the dead ones. Hence, the primary objectives of this study were to evaluate the potential of deep convolutional neural networks to distinguish between viable and non-viable *Eimeria* oocysts by capturing the presence/absence of granular structure in addition to other morphological changes using simple white light microscopy, such as phase-contrast (PC), differential interference contrast (DIC), and brightfield (BF) imaging. We sought to determine an optimal imaging method that enables deep learning-based discrimination of different viability stages of *Eimeria* oocysts. Additionally, we assessed the cross-species applicability of the deep learning model by testing the model trained to predict the viability of *E. acervulina* on other economically significant species, including *E. maxima* and *E. tenella*. Industry relevance requires that such a tool be applied to multiple species of *Eimeria*. Thus, we sought to determine suitable simple white light microscopy techniques and dataset characteristics that facilitate a deep learning-based evaluation of *Eimeria* oocyst viability. Our successful model advances a practical solution for achieving precise dosing of *Eimeria* vaccine with viable oocysts, facilitating better management of poultry coccidiosis. This approach also provides a tool to aid risk assessment and mitigation of foodborne pathogens like *Cyclospora*.

## Materials and methods

### *Eimeria* strains

Ten species of *Eimeria* cause most coccidiosis in chickens (Blake, 2025). Chickens infected with various *Eimeria* species may display different clinical or subclinical symptoms due to significant variations in pathogenicity and virulence among these species. Here, we focused on three specific species: *E. acervulina* (strain APU1), *E. tenella* (strain APU1), and *E. maxima* (strain APU1). These APU1 strains are laboratory-maintained strains held at the Animal Parasitic Diseases Laboratory, Agricultural Research Service, USDA (Beltsville, MD), where they are routinely propagated in straight-run HR308 or HR708 Hubbard-Ross chickens. These three species differ in their microscopic morphological features and oocyst sizes. Thus, developing a rapid viability prediction model using one of the species and translating it to others reflects the potential for generalization of the model.

We propagated oocysts through male Hubbard-Ross chickens every three months to ensure a consistent supply of viable oocysts for subsequent experiments (Blake et al., 2021; Proszkowiec-Weglarz et al., 2020; Ryley et al., 1976). Oocysts were sporulated for 24 to 48 hours with aeration provided by an aquarium pump and incubated at 29°C in a shaking water bath (FisherScientific, Hampton, NH). After sporulation, oocysts were preserved in a 2.0 % K_2_Cr_2_O_7_ solution at 4°C. An aliquot of each *Eimeria* species' oocysts was collected immediately (at 0 months) and then approximately every four months thereafter. Oocysts were concentrated by centrifugation at 1,000× g for three minutes, followed by two washes with sterile PBS. They were then resuspended in sterile PBS before undergoing microscopic analysis.

### Imaging and image processing

To develop a deep learning-based model to detect and identify the viability of *Eimeria* oocysts, we collected microscopic images of *E. acervulina, E. tenella*, and *E. maxima* using three imaging methods: phase-contrast (PC), differential interference contrast (DIC), and brightfield (BF). For PC imaging, a microscope (Axioskop 2, Zeiss, Dublin, CA) equipped with a 100x/1.3 oil h3 PC objective (Olympus, Center Valley, PA) was used. For DIC and BF imaging, we employed a microscope (AXIO Imager M2, Zeiss) equipped with a 63x/1.4 oil DIC objective and a 63x/1.4 oil BF objective. Table 1 summarizes the datasets used to develop our viability determination model. Viability of *Eimeria* oocysts was evaluated using autofluorescence flow cytometry with 488 nm laser excitation, validated by infection trials in broiler chicks. Broiler chicks were orally challenged with oocyst fractions separated according to their 488 nm-excited autofluorescence intensity, and oocyst shedding and clinical responses were monitored to link autofluorescence patterns to viability status: low-autofluorescence oocysts remained infective (live sporulated), high-autofluorescence oocysts were non-infective (dead sporulated), and intermediate signals corresponded to unsporulated oocysts (Valente et al., 2025). Using this 488 nm autofluorescence–based method, oocysts in the microscopic images were classified into the three viability categories for deep learning model training and evaluation. Images that were out of focus, contained overlapping oocysts, or were obscured by debris were screened out by an experienced parasitologist so that only high-quality images were retained for model training.

**Table 1:** Datasets used for deep learning-based detection of *Eimeria* oocysts.

Imaging method^1^	*Eimeria* species	Total number of images	Purpose
PC	*E. acervulina*	400	Determine an imaging method for deep learning-based detection
DIC		400	
BF		400	
PC	*E. acervulina*	600	Develop a deep learning-based detection model for *E. acervulina*
	*E. tenella*	200	Evaluate cross-species detection using a model trained on *E. acervulina*
	*E. maxima*	600	Evaluate species-specific detection after model fine-tuning on *E. maxima*

1 PC: phase-contrast; DIC: differential interference contrast; BF: brightfield

Preprocessing of the collected microscopic images was conducted using image processing tools available in Matlab (Mathworks Inc., Natick, MA). The images, initially in TIF format, were resized and converted into 640×640 pixels JPG format. Ground truth annotations for the images of *Eimeria* oocysts were performed by a trained microbiologist. We created bounding boxes around the oocysts, indicating their location and viability status, using the open-source Computer Vision Annotation Tool (Intel, Santa Clara, CA). Annotations were saved in XML format for subsequent processing. To evaluate the size of *E. acervulina* and *E. maxima*, the width and length of 30 oocysts of each species were measured using Image Processing Toolbox (Matlab).

### Model architecture and training details

Utilizing the PyTorch library in Python (Paszke et al., 2019), we built a classification model to assess the viability of *E. acervulina, E. tenella,* and *E. maxima* oocysts. This model incorporates the architecture of the YOLOv7 network (Wang et al., 2023), pretrained on the MS COCO 2017 dataset (Lin et al., 2014). First, we split the preprocessed images of *E. acervulina* oocyst for each class into a training dataset (70 %), a validation dataset (15 %), and a test dataset (15 %). To enhance the efficiency of model training, we applied various data augmentation techniques to the training images, including random horizontal and vertical flips, rotation, and variation in HSV (Hue, Saturation, and Value). A stochastic gradient descent optimizer was used with a weight decay of 0.0005, a momentum of 0.9, and a batch size of 4. Few used a validation dataset to optimize the hyperparameters of the model to ensure optimal training outcomes. The training process was performed on a high-performance computing setup, utilizing an NVIDIA A100 40GB GPU for effective model training. For the detection and classification of *E. maxima* oocysts, 200 PC images of each class (sporulated, unsporulated, and dead) were split into training (70 %), validation (15 %), and test (15 %) (Table 1). We used the split dataset of *E. maxima* to fine-tune the classification model originally trained on *E. acervulina* oocysts, using the same training settings, including data augmentation techniques, hyperparameter settings, and batch size.

### Model performance

After training the oocyst detection model, the model's performance was evaluated using the following metrics using a separate dataset.

(1) $$Precision=\;\frac{TP}{TP+FP}$$

(2) $$Recall=\;\frac{TP}{TP+FN}$$

where *TP, FP*, and *FN* are true positive, false positive, and false negative, respectively (Sultana et al., 2020).

## Results and discussion

Comparative Morphology of *E. acervulina* Oocysts Using Different Imaging Methods

**Fig. 1:**
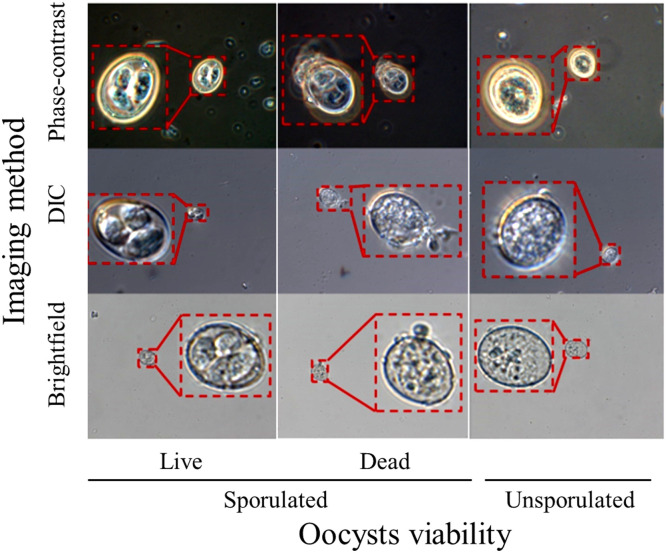
Representative images of *Eimeria acervulina* oocysts at different viability stages captured using phase-contrast, differential interference contrast, and brightfield imaging. Phase contrast images were captured using a microscope (Axioskop 2, Zeiss, Dublin, CA) equipped with a 100x/1.3 oil h3 PC objective (Olympus, Center Valley, PA), whereas DIC and BF imaging were performed using a microscope (AXIO Imager M2, Zeiss) equipped with a 63x/1.4 oil DIC objective and a 63x/1.4 oil BF objective, respectively. Red rectangles indicate the zoom version of the inserted images.

When first excreted, coccidian oocysts encompass an undifferentiated sporoblast, which subsequently mature to the infectious state, defined by four sporocysts. Four such sporocysts, each containing two sporozoites, characterize mature oocysts of species of *Eimeria.* Their sporozoites can invade host cells, establishing infection. We observed these morphological characteristics in *E. acervulina* using three imaging methods: phase contrast (PC), differential interference contrast (DIC), and bright field (BF) microscopy (Fig. 1). Variation among individual oocysts complicates their manual classification as dead or alive by means of subtle, but consistent differences. Characteristic features of dead, sporulated oocysts (structural breakdown defined by granular structures and ruptured oocyst walls) are not always clearly visible. These observations highlight the difficulty of reliable manual assessment, motivating the development of a deep learning-based image analysis approach designed to distinguish viable and non-viable oocysts despite subtle morphological variations.

Each imaging method provided unique insights into oocyst morphology. PC imaging, by enhancing contrast in translucent specimens (Murphy and Davidson, 2012), accentuated internal structures, facilitating morphological analysis of sporocysts and protoplasm within the oocysts (Fig. 1). DIC, which utilizes polarized light and prisms to highlight fine structural details and provide a sense of depth (Murphy and Davidson, 2012), enabled visualization of surface and internal structures with enhanced contrast and depth. BF microscopy, the simplest imaging method used in this study, passes light directly through the specimen; it achieves contrast by absorption of light in dense areas (Murphy and Davidson, 2012). Although BF provided less internal detail than PC or DIC, it proved useful for assessing oocyst shape and structure.

### Impact of imaging methods on deep learning-based detection of *E. acervulina* oocysts

To differentiate between sporulated oocysts from unsporulated oocysts and to distinguish live from dead sporulated oocysts, we employed YOLOv7, a deep learning-based object detection and classification algorithm (Wang et al., 2023). To assess which imaging method best supported this approach, we divided images of live and dead oocysts for each imaging method into three subsets: 70 % for training, 15 % for validation, and 15 % for testing. During training, the validation dataset was used to optimize the hyperparameters. A separate test dataset was then utilized to evaluate the model's performance on unseen data. As shown in Table 2, the deep learning model proved effective at classifying *E. acervulina* sporulated oocysts as live or dead based on their morphology, including presence or absence of granular structure (Valente et al., 2025), achieving overall precision and recall values above 87.4 % and 77.1 %, respectively (regardless of imaging method). The model trained with PC images nonetheless outperformed those trained with DIC and BF images (Table 2). Specifically, the overall precision and recall for PC images were 93.1 % and 91.2 %, respectively, compared to 84.1 % precision and 86.7 % recall for DIC images, and 87.9 % precision and 85.3 % recall for BF images. Thus, PC imaging supported superior performance, likely by enhancing contrast and visualization of internal structures. We therefore further evaluated PC images to elaborate on the potential of deep convolutional neural networks to detect and identify viable *E. acervulina* oocysts.

**Table 2:** Performance metrics for *Eimeria acervulina* oocyst detection using the deep convolutional neural networks with different imaging methods.

	Phase-contrast		Differential interference contrast		Brightfield	
	Precision	Recall	Precision	Recall	Precision	Recall
Live	93.1 %	91.2 %	84.1 %	86.7 %	87.9 %	85.3 %
Dead	91.2 %	88.6 %	92.3 %	79.9 %	87.0 %	69.0 %
Overall	92.1 %	90.8 %	88.2 %	83.3 %	87.4 %	77.1 %

### Dataset refinement for enhanced detection of live and dead *E. acervulina* oocysts by enriching the dataset with unsporulated oocysts

**Fig. 2:**
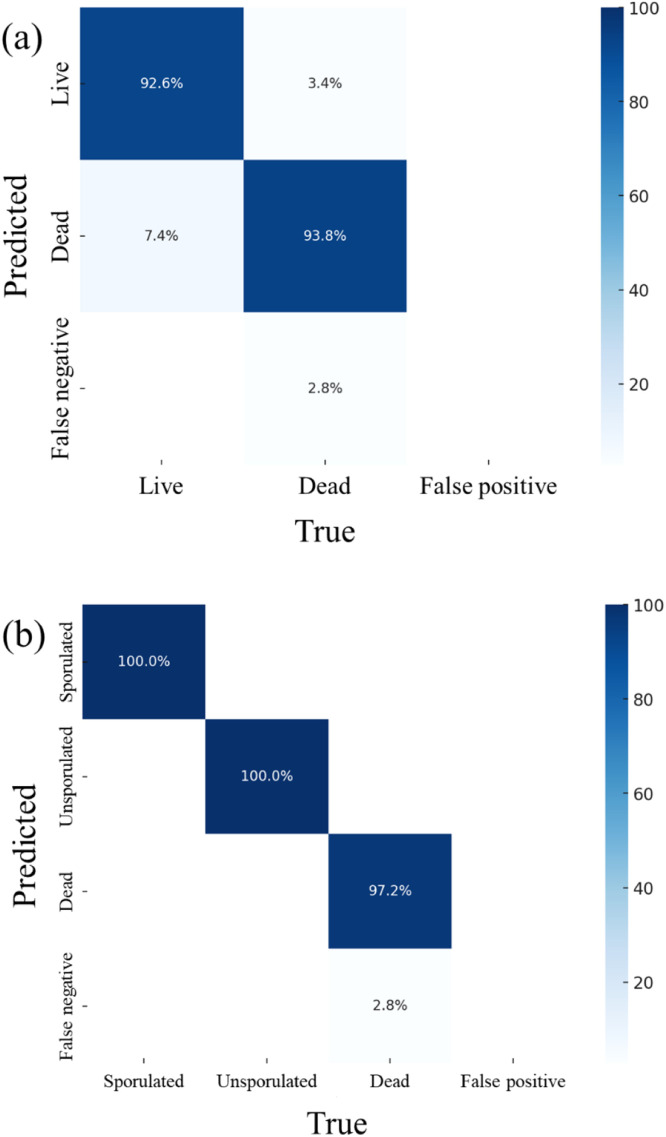
Detection of *Eimeria acervulina* oocysts using phase-contrast imaging and a deep convolutional neural network based on the YOLOv7 architecture: (a) results of 2-class classification (live and dead oocysts), and (b) results of 3-class classification (sporulated, unsporulated, and dead oocysts). Images were acquired using phase-contrast microscopy (Axioskop 2, Zeiss, Dublin, CA; 100×/1.3 oil h3 PC objective, Olympus, Center Valley, PA).

Among the population of oocysts, we had experimentally induced to sporulate using optimized conditions (Tucker et al., 2021), a portion (10 to 30 %) remained unsporulated. Such unsporulated oocysts might impede classification performance of the model, contaminating the “sporulated” training set with unsporulated oocysts (Langenkämper et al., 2018). To further improve model performance, we added 200 additional PC images of unsporulated oocysts to retrain the model. As shown in Fig. 2(a), doing so significantly improved classification performance; overall precision increased from 92.1 % to 94.6 %, and recall increased from 90.8 % to 94.9 % (Table 1).

Dead, sporulated oocysts accumulate granular structures, ruptured oocyst walls, fragmented sporocysts, and remnants of undifferentiated protoplasm (depending on their state of maturity when they died) (Fig. 1). Thus, to better train the deep learning model, we used such information to label oocysts as live sporulated, dead sporulated, and unsporulated oocysts (Fig. 2b). This 3-class system significantly outperformed the 2-class model described above. Thus, more specific labeling aided the deep learning model in discriminating among forms of *E. acervulina* oocysts (Fig. 2).

### Cross-species applicability of the oocyst detection model

**Fig. 3:**
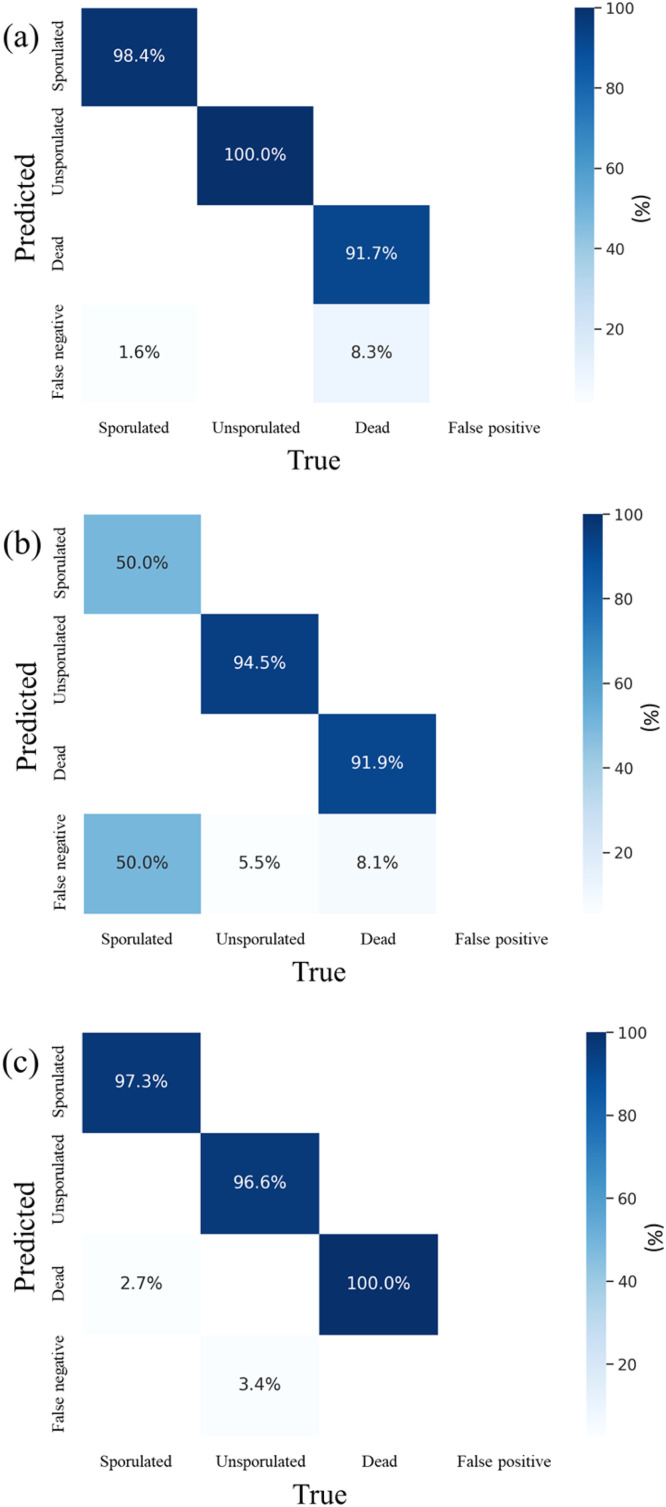
Cross-species detection of *Eimeria* oocysts using phase-contrast imaging and a deep convolutional neural network based on the YOLOv7 architecture: (a) results for *E. tenella* oocysts using the model trained on *E. acervulina*, (b) results for *E. maxima* oocysts using the model trained on *E. acervulina*, and (c) results for *E. maxima* oocysts after fine-tuning the model with an *E. maxima* dataset. Images were acquired using phase-contrast microscopy (Axioskop 2, Zeiss, Dublin, CA; 100×/1.3 oil h3 PC objective, Olympus, Center Valley, PA).

**Fig. 4:**
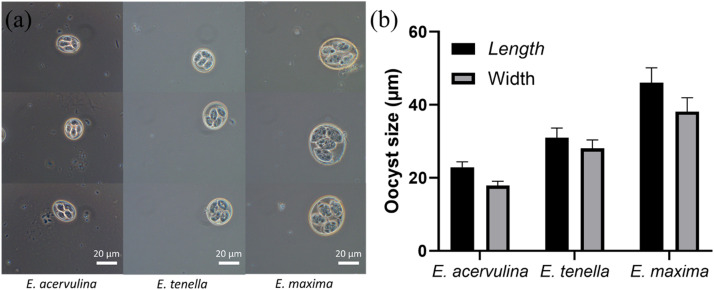
(a) Representative phase-contrast microscopic images and (b) size measurements of *Eimeria acervulina, E. tenella*, and *E. maxima* oocysts. Images were captured using phase-contrast microscopy (Axioskop 2, Zeiss, Dublin, CA; 100×/1.3 oil h3 PC objective, Olympus, Center Valley, PA), and size measurements were performed by image analysis.

Other species of *Eimeria* burden poultry health. Important among these include *E. maxima* and *E. tenella* (Liu et al., 2023; Williams, 2005). Indeed, infections with *E. maxima* and *E. tenella* more typically induce severe clinical disease than do infections with *E. acervulina*. Most live-attenuated vaccines (i.e. Evant, Evalon, and Coccivac-D2 and B-52) therefore include *E. acervulina, E. maxima*, and *E. tenella*. We therefore sought to understand the power of our model, trained on oocysts of *E. acervulina,* to determine viability of oocysts of *E. maxima* and *E. tenella*. Notably, the model achieved 100 % overall precision and 98.1 % overall recall for *E. tenella* (Fig. 3a). Such success no doubt derives from their notable morphological similarities (comparable size and similar sporocyst morphology) (Conway and McKenzie, 2007) (Fig. 4).

The model proved less capable of determining viability for oocysts of *E. maxima* (overall recall of 43.5 %) (Fig. 3b). Poorer performance likely derives from the considerably larger size of *E. maxima* oocysts (Fig. 4a). *E. maxima* measured 46.1 ± 4.1 µm × 38.1 ± 3.8 µm, compared to *E. acervulina* (22.9 ± 1.5 µm × 17.9 ± 1.2 µm) and *E. tenella* (31.0 ± 2.6 µm × 28.1 ± 2.3 µm) (*p* < 0.05) (Fig. 4b). To improve performance on oocysts of *E. maxima*, we fine-tuned the model using 600 images of *E. maxima* (200 images each of sporulated live and dead, and unsporulated oocysts). This achieved classification accuracy of over 95 % (Fig. 3c). Thus adjusted, the oocyst detection model accurately detected and classified oocysts from all three *Eimeria* species. Oocyst size notwithstanding, the model then identified shared changes related to viability state. Thus, deep convolutional neural networks, based on the YOLOv7 architecture, can effectively identify the viability of a wide range of *Eimeria* oocysts, imaged with simple phase-contrast microscopic images. Success requires neither sophisticated instruments nor extensive sample preparation.

### Potential benefits to the poultry and food industries

Deep learning-based image analysis methods, efficacious for classifying foodborne bacteria and yeast (Chen et al., 2024; Kang et al., 2024; Kim et al., 2021; Ma et al., 2023; Park et al., 2025a; Park et al., 2025b; Yi et al., 2023; Quan et al., 2024), have seldom been applied to detect and classify coccidian parasites. To date, none have leveraged such tools to assess parasite viability. For example, Li et al. (2019) utilized a convolutional neural network model to detect *Eimeria* parasites in microscopic images, achieving an accuracy of 92 % (compared to manual evaluation). Smith et al. (2023) developed an AI-based automated image analysis model to rapidly detect and enumerate different sizes of *Eimeria* oocysts. Nagamori et al. (2021) used a model based on YOLOv3 to classify *Ancylostoma* eggs, *Toxocara cati* eggs, *Cystoisospora* oocysts, and *Giardia* cysts obtained from fecal samples. They achieved a sensitivity range of 75.8 %-100 %. Boufenar et al. (2022) and He et al. (2023) applied machine learning algorithms to discriminate among 7 different *Eimeria* species, achieving greater than 97 % accuracy.

Assessing viability aids in risk assessment and can ensure that vaccine preparations will prove efficacious (Jenkins et al., 2023; Snyder et al., 2023). Thus, the model developed here leverages simple white light microscopy to provide an effective and practical tool for the poultry industry. Competing methods require sophisticated instruments such as PCR (Liang and Keeley, 2012; Rousseau et al., 2019) or flow cytometry (Adams et al., 2022; Jang et al., 2014; Robinson et al., 2023). Extensive staining processes and specialized expertise impede widespread adoption of those alternatives. In addition to supporting vaccine formulation, the proposed approach may also benefit the food industry by enabling rapid screening of viable protozoan contaminants such as *C. cayetanensis* in fresh produce. Given the morphological and developmental similarity between *Eimeria* and *Cyclospora*, the deep learning-based approach could aid in evaluating sanitation efficacy and microbial risks in produce processing environments, which offers a cost-effective and accessible solution suitable for routine monitoring in both poultry and food sectors.

## Conclusion

We achieved a deep learning-based image processing method to evaluate the viability of *Eimeria* oocysts. This approach offers a practical means to ensure consistent vaccine formulation, improving coccidiosis management in the poultry industry to the benefit of animal health and producer profitability. This approach initially distinguished live from dead *E. acervulina* oocysts with a precision of 94.6 % and a recall of 94.9 %. Further refinements improved performance to 99.1 % precision and recall to 99.1 %. We further trained the model to discriminate viable oocysts in species varying considerably in oocyst size, achieving accurate assessment for *E. acervulina, E. tenella,* and *E. maxima*. Unlike traditional viability assessment methods that require specialized equipment and staining processes, our approach offers a simple and cost-effective alternative suitable for routine monitoring. In addition to helping ascertain vaccine efficacy, this approach offers promise to support risk assessment programs by simplifying and accelerating assessment of these and related parasites such as *Cyclospora cayetanensis*.

## CRediT authorship contribution statement

**Hyeon W. Park:** Writing – original draft, Validation, Investigation, Formal analysis. **Matthew J. Valente:** Writing – review & editing. **Valsin Fournet:** Writing – review & editing. **Benjamin M. Rosenthal:** Writing – review & editing. **Mark Jenkins:** Writing – review & editing. **Asis Khan:** Funding acquisition, Writing – review & editing, Supervision, Conceptualization. **Nitin Nitin:** Writing – review & editing, Supervision, Funding acquisition, Conceptualization.

## Disclosures

The authors declare no competing interests.
